# Antioxidant Interactions between Citrus Fruit Carotenoids and Ascorbic Acid in New Models of Animal Cell Membranes

**DOI:** 10.3390/antiox12091733

**Published:** 2023-09-07

**Authors:** Marcelo P. Barros, Jaime Zacarías-Garcia, Florencia Rey, Lorenzo Zacarías, María J. Rodrigo

**Affiliations:** 1 Food Biotechnology Department, Institute of Agrochemistry and Food Technology, Spanish National Research Council (IATA-CSIC), Catedrático Agustín Escardino 7, Paterna, 46980 Valencia, Spain; jaizagar@iata.csic.es (J.Z.-G.); floreyrob@iata.csic.es (F.R.); lzacarias@iata.csic.es (L.Z.); 2 Interdisciplinary Program in Health Sciences, Institute of Physical Activity Sciences and Sports (ICAFE), Cruzeiro do Sul University, Rua Galvao Bueno 868, São Paulo 01506-000, Brazil

**Keywords:** free radicals, oxidative stress, lipid peroxidation, micronutrient, singlet oxygen, nutrition, food, vitamin

## Abstract

The regular consumption of citrus fruits by humans has been associated with lower incidence of chronic-degenerative diseases, especially those mediated by free radicals. Most of the health-promoting properties of citrus fruits derive from their antioxidant content of carotenoids and ascorbic acid (ASC). In the current work we have investigated the scavenging (against hydroxyl radical) and quenching capacities (against singlet oxygen) of four different carotenoid extracts of citrus fruits in the presence or absence of ASC (μM range) in organic solvent, aqueous solution, micelles and in an innovative biomimicking liposomal system of animal cell membrane (AML). The fruits of four varieties of citrus were selected for their distinctive carotenoid composition (liquid chromatography characterization): ‘Nadorcott’ mandarin and the sweet oranges ‘Valencia late’, ‘Ruby Valencia’ and ‘Pinalate’ mutant. The quenching activity of citrus carotenoids strongly depended on the biological assemblage: freely diffusible in organic solvent, ‘Ruby Valencia’ carotenoids (containing lycopene) showed the highest quenching activity, whereas ‘Nadorcott’ mandarin extracts, rich in β-cryptoxanthin, prevailed in micellar systems. Interestingly, the addition of 10 μM ASC significantly increased the quenching activity of all citrus extracts in micelles: ‘Valencia’ orange (+53%), ‘Pinalate’ (+87%), ‘Ruby’ (4-fold higher) and ‘Nadorcott’ mandarins (+20%). Accurate C_11_-BODIPY^581/591^ fluorescence assays showed solid scavenging activities of all citrus extracts against AML oxidation: ‘Valencia’ (−61%), ‘Pinalate’ (−58%) and ‘Ruby’ oranges (−29%), and ‘Nadorcott’ mandarins (−70%). Indeed, all four citrus extracts tested here have balanced antioxidant properties; extracts from the ‘Nadorcott’ mandarin slightly prevailed overall, due, at least in part, to its high content of β-cryptoxanthin. This study depicts some of the antioxidant interactions between citrus fruit carotenoids and ascorbic acid in models of animal cell membranes and reinforces the contribution of them in promoting health benefits for humans.

## 1. Introduction

Nutritionists and health professionals have long been suggesting that a “colorful diet” is a synonym for good health [1]. Indeed, most of the naturally pigmented foods contain important micronutrients for human health, like vitamins, flavonoids, carotenoids, and anthocyanins [2].

Citrus fruits and their juices are probably one of the most accessible and relevant sources of key antioxidants for humans worldwide [3]. Apart from their significant content of ascorbic acid and flavonoids, the antioxidant properties of citrus fruits strongly depend on the carotenoid composition in different species such as oranges, mandarins and grapefruits [4]. The most consumed citrus as fresh fruit are mandarins and oranges, which show contrasting carotenoid profiles between them. In fact, β,β-xanthophylls prevail in the pulps of both sweet oranges and mandarins. However, 9-Z-violaxanthin is actually the main carotenoid in ordinary or standard oranges, while mandarins (and their hybrids) have an elevated proportion of β-cryptoxanthin, a xanthophyll provided with provitamin A activity [5]. Other carotenes, such as phytoene, phytofluene and β-carotene, and the xanthophylls lutein, zeaxanthin and antheraxanthin are regularly present in the pulp of most of the varieties of sweet oranges (*Citrus sinensis*) and mandarins (*Citrus reticulata*), but in minor proportions [6]. Different varieties of sweet oranges have been described with altered fruit pigmentation and carotenoid composition. The sweet orange ‘Pinalate’ is a spontaneous bud mutation derived from the navel orange ‘Navelate’, which has a yellow-colored pulp due to the overaccumulation of the colorless or yellow carotenes, phytoene, phytofluene and ζ-carotene, with reduced levels of β,β-xanthophylls [7,8]. The red pigmentation of citrus fruit pulp is an attractive trait for consumers and several red-fleshed sweet orange varieties have been selected in breeding programs and characterized, such as the recently described ‘Kirkwook Navel’ and ‘Ruby’ Valencia, both of South African origin [9]. ‘Navel Kirkwood’ and ‘Ruby’ Valencia have a similar carotenoid profile in the flesh, with high phytoene and phytofluene contents and a moderate accumulation of lycopene, which gives them their attractive red pulp color [9]. 

Evidence shows that most of the health benefits provided by carotenoid-rich diets are associated with carotenoid contents, the proportion between xanthophylls and carotenes and possible synergism with other antioxidants [10,11,12]. Regarding biochemical mechanisms, the molecular interactions between carotenes and xanthophylls and their spatial distribution within lipid bilayers in target cells might explain the enhanced antioxidant properties of mixed xanthophyll/carotenes in extracts [13]. Carotenoids are recognized as powerful antioxidants by both intercepting free radicals in lipid bilayers (scavenging activity) and suppressing or quenching the excitatory energy of singlet oxygen, [O_2_(^1^Δ_g_)] [14]. On the other hand, many studies have already reported the pro-oxidant effects of carotenoids and ascorbic acid under specific—sometimes unpredictable—microenvironmental conditions, which reinforces the importance of understanding the interplay mechanisms of these compounds against reactive oxygen/nitrogen species (ROS/RNS) [15,16].

Based on that, we aimed here to investigate the antioxidant properties of the micromolar concentrations (μM) of ascorbic acid and carotenoid extracts from citrus fruits in animal cell membrane models. For that purpose, we selected the pulp of fruits of four citrus varieties with contrasting carotenoid content and composition: a highly pigmented mandarin (*Citrus reticulata*) variety ‘Nadorcott’ [17,18] with a predominance of β,β-xanthophylls and elevated content of β-cryptoxanthin [4]; and three varieties of sweet orange (*Citrus sinensis*): ‘Valencia late’, a standard orange-pigmented variety with moderate contents of β,β-xanthophylls, 9-Z-violaxanthin being the main carotenoid [4,9,19]; ‘Ruby’ Valencia, a red-fleshed orange with a very high concentration of non-polar carotenes phytoene and phytofluene and moderate concentration of lycopene and β,β-xanthophylls [4,9,19]; and ‘Pinalate’, a sweet orange mutant with a pale yellow-pigmented peel and pulp, accumulating the colorless carotene phytoene and phytofluene and a reduced content of β,β-xanthophylls [20,21]. For a more realistic model of antioxidant effects in human cells, we report here the development of an innovative biomimicking system for the animal cell membrane, which was evaluated pursuant to its oxidizable capacity as a target for lipid peroxidation. The antioxidant properties of the four carotenoids extracts, with/without ascorbic acid, were evaluated by their scavenging activities against hydroxyl radical (HO^•^) produced in vitro and their quenching activity against singlet oxygen [O_2_(^1^Δ_g_)] in organic solvent and in micellar systems. This multilayered approach facilitates comprehension on how the water-soluble ascorbic acid (ASC) interacts with lipid-soluble carotenoids, both present in citrus fruits, to improve the antioxidant protection of cell membranes, which is currently conceived as one of the molecular mechanisms responsible for good health in humans. 

## 2. Materials and Methods

### 2.1. Chemicals

All chemicals were obtained from Sigma-Aldrich^®^ (Madrid, Spain), except liquid chromatography grade solvents *n*-hexane, chloroform, dichloromethane (DCM), methanol (MeOH) and ethanol (EtOH), which were purchased from Scharlab (Barcelona, Spain) and methyl *tert*-butyl ether (MTBE) from Merck (Darmstadt, Germany). The fluorescent probe C_11_-BODIPY^581/591^ was purchased from Invitrogen^TM^ (Waltham, MA, USA). The singlet oxygen [O_2_(^1^Δ_g_)] generator 1,4-naphthalene endoperoxide (EP) was purchased from InvitroTech^®^ (Kyoto, Japan).

### 2.2. Plant Material and Storage Conditions

All fruits were harvested at commercial maturity from adult trees growing under standard Mediterranean agronomical and environmental conditions. ‘Nadorcott’ mandarins were harvested in February 2017 from a commercial orchard located in Lliria (Valencia, Spain). The sweet orange varieties ‘Valencia late’ and ‘Ruby’ Valencia were harvested in March 2017 from the Fundación ANECOOP (Museros, Valencia, Spain). Fruit of the ‘Pinalate’ sweet orange mutant were harvested in February 2017 from the Citrus Germplasm Bank (IVIA, Instituto Valenciano de Investigaciones Agrarias, Moncada, Valencia, Spain).

Approximately 30 fruits of each genotype were harvested from the external tree canopy and immediately delivered to the laboratory. Fruits were sliced into halves and the pulp tissue was excised in small cubic pieces (of approximately 1 cm^3^) containing juice vesicles and free of segment membranes. Juice vesicle pieces were immediately frozen in liquid nitrogen, ground to powder with liquid nitrogen with an electric mill and stored in freezer at −80 °C until analysis.

### 2.3. Carotenoid Extracts

Carotenoids were extracted from frozen samples of citrus fruit pulp in organic solvents as previously described [22]. Briefly, aliquots (2 g of fresh weight; FW) of frozen pulp samples of each citrus variety were weighed in screw-capped glass tubes, and 4 mL of chromatography-grade MeOH plus 3 mL of the buffering solution Tris-HCl 50 mM pH 7.5 containing 1 M NaCl were added. Four mL of chloroform were added, and the mixture was sonicated 5 min in XUBA3 ultrasonic water bath (Grant Instruments, England). After that, samples were centrifuged at 3000× *g* for 10 min at 4 °C. The organic phase containing carotenoids was collected in foil-wrapped glass tubes to avoid photodegradation. Additional washes of the upper phase and pulp tissue residue were conducted by adding 4 mL of DCM vortexing, centrifugation at 3000× *g* for 10 min at 4 °C and organic phases were pooled together. This procedure was repeated at least four times or until the organic phase was colorless to assure complete carotenoid extraction from pulp tissue. After that, the collected organic extract was dried on a rotatory evaporator at 30 °C, and a thin layer of total carotenoids from samples was formed on round-bottom flasks. Samples were then saponified in methanolic KOH solution (6% *w*/*v*) overnight at room temperature. Saponified carotenoids were recovered from the upper phase after adding water and petroleum ether:diethyl ether (9:1) to the mixture. Carotenoid extracts were dried under nitrogen stream and kept at −80 °C until high-performance liquid chromatography (HPLC) analysis or preparation of liposomes.

### 2.4. Carotenoid Quantification

The carotenoid extracts were characterized and quantified by high-performance liquid chromatography-diode array detection (HPLC-DAD), which scans the entire UV-visible light spectrum during analysis, as described by Rodrigo et al. (2015) [22]. The liquid chromatography system was equipped with a 600E pump, a DAD model 2998 and Empower3 software (Waters^®^, Barcelona, Spain). A C_30_-carotenoid column (250 × 4.6 mm, 5 μm) coupled to a C_30_ guard column (20 × 4.0 mm, 5 μm) (YMC, Teknokroma, Spain) was used. Samples were prepared for HPLC by dissolving the dried carotenoid extracts in chloroform:MeOH:acetone (3:2:1, *v*:*v*:*v*). A ternary gradient elution with MeOH, water and MTBE was used for carotenoid separation [8,10,23]. The carotenoids were identified by absorbance spectra and retention time. For each elution, a Maxplot chromatogram was obtained which integrated each carotenoid peak at its corresponding maximum absorbance wavelength and their contents were calculated using the appropriate calibration curves, as described elsewhere [8,10,23].

### 2.5. Carotenoid and Ascorbic Acid Stock Solutions

Dried citrus carotenoid extracts obtained as described in 2.3 (Carotenoid extracts) were stored in 5 mL-glass tubes at −80 °C in an ultrafreezer, protected from light and under a nitrogen atmosphere to prevent oxidation and isomerizations. To prepare the aliquots for assays, proper volumes of chloroform were added to the dried extract to obtain standard 10 mM solutions of carotenoids. Carotenoid concentration in the extracts was calculated using the average molecular mass weight of carotenoids as 550 g/mol. After use, the organic solvent was removed once again by N_2_ flow at room temperature and the dried carotenoid stocks under nitrogen flow stored at −80 °C. Analytical grade ascorbic acid (ASC) was used to prepare a 20 mM standard solution in phosphate saline buffer 50 mM, pH 7.5, for oxidation assays.

### 2.6. Experimental Design

**Figure 1:**
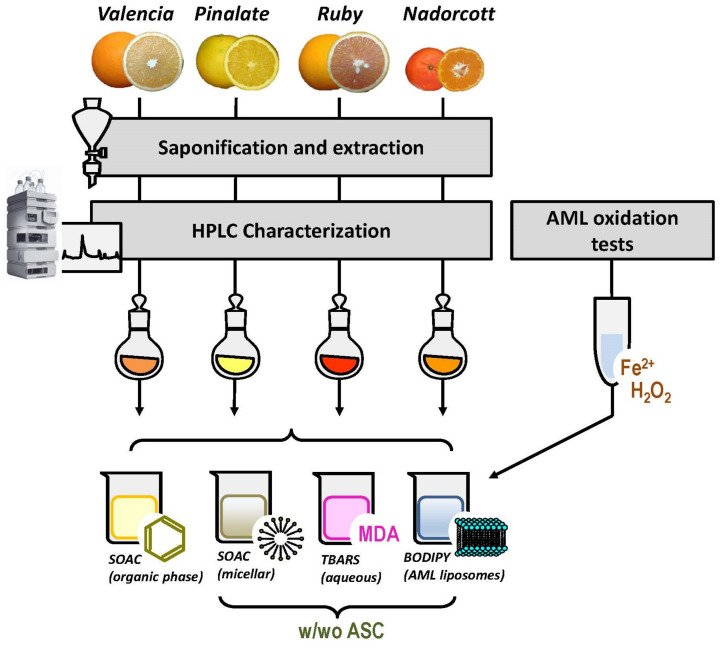
Experimental design of the study. w/wo ASC: with and without ascorbic acid.

The antioxidant capacities of citrus carotenoids, in the presence or absence of ascorbic acid (ASC), were determined in four different systems: (i) organic solvent, we tested here the inherent capacity of citrus fruit carotenoids to quench singlet oxygen [O_2_(^1^Δ_g_)] with the classic method of singlet oxygen absorption capacity (SOAC) [24]; (ii) micellar system, in order to investigate a possible quenching interplay between citrus carotenoids and the water-soluble ASC, the SOAC method was adapted for Triton X-100 micellar systems, as described by Mukai et al. (2017) [25]; (iii) liposomal system, after preliminary assays to describe the oxidizable properties of the novice animal cell membrane biomimicking liposomes (AML), carotenoid-loaded AML were used as targets for lipid peroxidation initiated by HO^•^ radicals (Fenton reaction). Lipid oxidation was monitored by fluorescence decay of the membrane-bound probe C_11_-BODIPY^581/591^ [26]; and (iv) aqueous solution, the classic method of thiobarbituric acid-reactive substances (TBARS) was used to measure lipid-derived aldehydes, including malondialdehyde (MDA), after AML exposure to HO^•^ radicals [27,28]. Figure 1 represents the experimental design of our study.

### 2.7. Determination of Singlet Oxygen Absorption Capacity (SOAC)

The procedure used for SOAC determination was described by Ouchi et al. (2010) with modifications described by Takahashi et al. (2016) [25,29]. Briefly, approximately 1.0 g of frozen pulp material was extracted in 6 mL of cooled EtOH/chloroform/water solution (ECW; 49:50:1, *v*/*v*/*v*) using a pre-chilled mortar and pestle on an ice bath with sea sand as an abrasive. The crude homogenate was first filtered and then centrifugated at 4500 rpm 4 °C for 5 min for debris removal. After that, samples were concentrated on a rotatory evaporator with N_2_ flow at 30 °C (temperature to minimize oxidation) and the total volume of samples was corrected to 300 μL (in ECW). We determined the concentration of total carotenoids in the SOAC extracts by measuring the absorbance of extracts in organic solvent at the λ_max_ of the dominant carotenoid and respecting the composition of extracts following HPLC characterization. It is worth mentioning that the carotenoid isolation procedure here is different from that presented for full HPLC carotenoid characterization in extracts; considering the basic principles of chemical extraction of carotenoids (or lipid fractions) with organic solvents from raw material, we do not expect to observe critical changes in carotenoid composition between both extracts. Moreover, minor contributions from lipid-soluble contaminants are not discarded. 

Using an average molecular mass of 550 g/mol for total carotenoid estimation, all SOAC assays contained 25 μM total carotenoids in the reaction system, which was composed by 1.5 mM EP (singlet oxygen generator) and 0.10 mM 2,5-diphenyl-3,4-benzofuran (DPBF, indicator of singlet oxygen). DPBF concentration in ECW was measured by absorbance at 413 nm for 60 min at 35 °C. Standard solutions of 1.8 mM and 3.6 mM of α-tocopherol were used to calculate the relative SOAC value as follows:

SOAC value = [αtoc]/[carotenoid] × (t_1/2_ sample − t_1/2_ blank)/(t_1/2_ αtoc − t_1/2_ blank αtoc) (1)

where: [αtoc], concentration of α-tocopherol, in mmol/L; [carotenoid], concentration of carotenoid, in mmol/L; t_1/2_ sample, time elapsed for 50% decrease in initial absorbance with samples in the reaction system; t_1/2_ blank, time elapsed for 50% decrease in initial absorbance in blank system; t_1/2_ αtoc, time elapsed for 50% decrease in initial absorbance with αtoc in the reaction system.

### 2.8. Determination of Micellar Singlet Oxygen Absorption Capacity (SOAC_mic_)

Micellar systems were here used to evaluate possible [O_2_(^1^Δ_g_)]-quenching interplays between the water-soluble antioxidant ASC and citrus carotenoids in micellar/lipid phases. Triton X-100 was selected as the surfactant for this study since all chemicals of this method (carotenoids, α-tocopherol, DPBF and EP) have higher solubility in it compared to other common surfactants, such as sodium dodecyl sulfate (SDS) and cetyltrimethylammonium bromide (CTAB) [25]. In addition, Triton X-100 is an uncharged compound at pH 7.5, while SDS and CTAB molecules have negative and positive charges, respectively. Therefore, a 5% Triton X-100 (*w*/*v*) micellar solution in 50 mM phosphate-buffered saline (PBS), pH 7.5, was prepared by strongly vortexing the surfactant with the PBS buffer solution (the formed foam was discarded). Samples and standards were prepared in chloroform to reach final concentrations of 10 μM citrus carotenoids, 10 μM and 20 μM α-tocopherol (primary standard), and 10 μM, 20 μM and 30 μM β-carotene (secondary standard). Proper volumes of carotenoids and standards were injected using a fine needle (microsyringe Hamilton 705 N, 50 μL) into 30 mL of a Triton X-100 micellar solution, as previously described. The solution was gently stirred in a water bath at 20 °C for at least 30 min and the organic solvent was removed by N_2_ flow. The reaction system was composed of 133 μM DPBF and 3.3 mM EP in Triton X-100 micellar solution. After reaction, 200 μL of samples were applied in a quartz microplate at ∼20 °C (to prevent decomposition of EP). Then, the equipment temperature was set for 35 °C for approximately 3 min to start EP decomposition and the UV−vis absorption spectra were registered for 90 min (λ_max_ = 413 nm). SOAC_mic_ values were calculated as described in Equation (1).

### 2.9. Fitting Curves

For the accurate calculation of the half-time indexes (t_1/2_) of SOAC and SOAC_mic_ kinetics, we used the algorithm 30.1.104. Logistic function in growth/sigmoidal systems was developed by the software OriginPro 2016, 64-bit, Sr-2. This algorithm adjusts the spectral data to a smoothed sigmoidal curve, defined by the formula:

y = A_2_ + (A_1_ − A_2_)/[1 + (x/x_0_)^p^] (2)

where: A_1_, initial value; A_2_, final value; x_0_, center (the t_1/2_ value); and p, power.

The fitting curves involved data points from t_0_ = 5 min to t = 90 min since it takes approximately 3 min for the microplate reader to reach 35 °C, which is the temperature that triggers the decomposition of EP for [O_2_(^1^Δ_g_)] formation. With this fitting adaptation, correlation indexes (R^2^) were all > 0.97, which confirms the accuracy of t_1/2_ determinations. The Supplementary Materials S1 illustrates sigmoidal fitting curves of (A) SOAC determination of some citrus carotenoid samples used here and (B) SOAC_mic_ determinations, including two blank reactions lacking the [O_2_(^1^Δ_g_)] generator, 3 mM EP (curves B and C1; Supplementary Materials S1B).

### 2.10. Liposomal Systems

Unilamellar liposomes that mimic the lipid composition of animal cell membranes (AML; 1.5 mM total lipid content) were prepared with 0.72 mM (48%) egg yolk phosphatidylcholine (PC), 0.30 mM (20%) non-esterified cholesterol, 0.24 mM sphingolipids (16%; mostly ceramides) and 0.24 mM (16%) egg yolk phosphatidylethanolamine (PE) [30]. AML liposomes were idealized to reproduce the water/lipid interface where carotenoids first perform their antioxidant activity against ROS/RNS in animal cell membranes [31]. Egg yolk PC and PE have a significant % of polyunsaturated fatty acids, which are major targets of the oxidative attack promoted by HO^•^ radicals and other ROS/RNS [32]. All lipid components were solubilized in chloroform (stock solutions with different mM concentrations) for short-term utilization in the following oxidation assays. Simpler 1.5 mM PC liposomes were also prepared for preliminary oxidation assays and comparison with AML systems. AML liposomes containing carotenoid extracts from citrus fruits (10 μM total carotenoids in 1.5 mM AML liposomes) were prepared by mixing proper volumes of carotenoid stock solutions with AML lipids (all solutions in chloroform), avoiding a carotenoid:lipid ratio >1.0% mol which increases the chances of carotenoid aggregate formation [33]. After preparing the carotenoid:lipid solutions, chloroform was removed by flushing N_2_ in a round-bottom flask adapted to a rotavapor apparatus working at a low speed and moderate heating (≤37 °C) to allow the formation of a homogeneous dried carotenoid:lipid film. The carotenoid:lipid film was stored overnight in the dark and under vacuum to eliminate traces of chloroform. Then, multilamellar liposomes were preliminarily prepared by strongly vortexing proper volumes of 50 mM phosphate-buffered saline solution (PBS 50 mM, pH 7.5) added to the lipid film for 5 min at room temperature. Ultrasonication was avoided to prevent AML liposomes from artifactual oxidation by heating or Ti^3+/4+^ ions releasing from the equipment horn [34].

Freshly made multilamellar liposomes were then used to prepare unilamellar AML liposomes by extrusion through 100 μm-pore polycarbonate membranes (MilliPore, Burlington, MA, USA) at 37 °C in a Mini-Extruder device (Avanti Lipids. Co., Alabaster, AL, USA). Through 15 passes, clean, transparent and homogeneous unilamellar AML and PC liposomes were obtained and immediately used in the oxidation assays. Control AML liposomes lack citrus carotenoids. AML liposomes were kept in water-ice bath during the preparation for the oxidation assays.

### 2.11. Preliminary Oxidation Assays

Preliminary oxidation assays were performed to study the spontaneous oxidation of AML and PC liposomes in atmospheric air. After that, PC and AML liposomes were also exposed to different concentrations of reactive hydroxyl radicals (HO^•^) formed in aqueous solution by the Fenton reaction (Equation (3)). These assays were necessary to determine the suitable degree of lipid peroxidation for an accurate analytical detection in our novice liposomal systems here. Based on the Fenton reaction, reactive HO^•^ radicals are formed in aqueous solution (PBS 50 mM, pH 7.5) by mixing appropriate concentrations of hydrogen peroxide (H_2_O_2_) and ferrous ions (Fe^2+^) chelated with ethylenediaminetetraacetic acid (EDTA) [35].

H_2_O_2(aq_._)_ + Fe^2+^_(aq_._)_ → OH^−^_(aq_._)_ + HO^•^_(aq_._)_ + Fe^3+^_(aq_._)_ (3)

After preliminary assays, we defined an oxidizing system composed of 25 mM H_2_O_2_, 1.5 mM Fe^2+^ and 6 mM EDTA (1:4 ratio Fe^2+^:EDTA) to generate a sufficient HO^•^ concentration for the extended lipid oxidation of liposomes in aqueous systems.

### 2.12. Liposome Oxidation

The progression of lipid oxidation in PC and AML liposomes was monitored by the fluorescence probe C_11_-BODIPY^581/591^ [26]. C_11_-BODIPY^581/591^ is very sensitive to oxidation by hydroxyl (HO^•^), peroxyl (ROO^•^) and alkoxyl radicals (RO^•^), all of them main initiation/propagation agents of lipid oxidation in biological membranes. The fluorescent probe C_11_-BODIPY^581/591^: (i) anchors its undecanoic acid group with high stability to natural or synthetic lipid bilayers; (ii) shows good spectral separation of the non-oxidized (595 nm) and oxidized (520 nm) forms; (iii) has a good photo-stability and displays very few fluorescence artifacts; (iv) is virtually insensitive to microenvironmental changes, such as pH or solvent polarity; (v) once oxidized, the probe remains lipophilic and does not spontaneously leave the lipid bilayer; and (vi) is comparably sensitive to oxidation of common unsaturated fatty acids of biological membranes [36]. 

Therefore, a 0.4 mM stock solution of C_11_-BODIPY^581/591^ in methanol was prepared and kept in freezer −20 °C for further use. The fluorescent probe was incorporated in the systems during the multilamellar step of liposome preparation (as mentioned before) to reach a final concentration of 10 μM in PC or AML liposomes. The kinetics of lipid oxidation were determined by the decay of C_11_-BODIPY^581/591^ fluorescence emission at 600 nm (λ_excit_. = 575 nm) for 150 min at 35 °C. At time zero (immediately after Fe^2+^ ions addition), fluorescence intensity was adjusted to 1.0 unit and, thus, hereafter defined as relative fluorescence intensity (arbitrary units, A.U.). Under these circumstances, we assumed a pseudo first-order reaction of free radicals with C_11_-BODIPY^581/591^, which is well-fitted by the 1st order exponential decay function (Equation (4)) [37]:

y = y_0_ + A_1_.e^(-kt)^ (4)

where: y = relative fluorescence intensity; y_0_ = relative fluorescence intensity at time zero (equals 1.0); A_1_ = attenuation factor; k = rate constant; t = time (min).

### 2.13. Thiobarbituric Acid-Reactive Substances (TBARS)

In addition, the extension of lipid oxidation was measured by the thiobarbituric acid-reactive substances assay (TBARS), which is quantitatively sensitive to lipid-derived aldehydes, such as malondialdehyde (MDA). Lipid-derived aldehydes are known endpoint products of lipid oxidation, especially from polyunsaturated fatty acids. The TBARS assay has been widely used in the literature and it works reasonably well when applied to isolated systems, such as liposomes and microsomes, although its application to body fluids, cells and tissue samples is arguably unreliable [38]. Due to the lower sensibility of the TBARS method compared to that using the C_11_-BODIPY^581/591^ fluorescent probe, the oxidizing capacity of the reaction system for TBARS assay was doubled to 50 mM H_2_O_2_, 3 mM Fe^2+^ and 12 mM EDTA, but keeping the same (1:4) ratio of Fenton reactants used before. The AML liposomes were oxidized for 60 min at 35 °C. After 60 min, the progressive oxidation reactions were immediately stopped by adding 8 mM butylated hydroxytoluene (BHT; solubilized in EtOH). TBARS measurements were performed as described by Fraga et al. (1988) with modifications [27,39]. Briefly, 300 μL of oxidized liposome samples were mixed with 50 μL of PBS 50 mM (pH 7.5) and 600 μL 0.35% thiobarbituric acid in 0.25 M HCl containing 2% Triton X-100 (*v*/*v*) and incubated at 80 °C, 15 min, for the formation of the pinkish chromophore. A gelatinous aggregate was dissipated by vigorous vortex for few seconds. If necessary, a quick centrifugation (5000 rpm, 5 min, at room temperature) was applied to remove insoluble debris in samples. Clean supernatants were isolated and their absorbances were measured at 535 nm using 1,1′,2,2′-tetraethoxypropane (TEP) as a standard.

### 2.14. Statistical Analysis

Data are presented as mean ± standard deviation, (x ± SDc) and the statistical analysis performed with the t-Student’s test at significance level of 5% (OriginPro 2016, 64-bit, Sr-2). Graphics were plotted with Excel 2016, Microsoft Office 365 and OriginPro 2016, 64-bit, Sr-2.

## 3. Results

### 3.1. Characterization of Carotenoid Extracts

**Figure 2:**
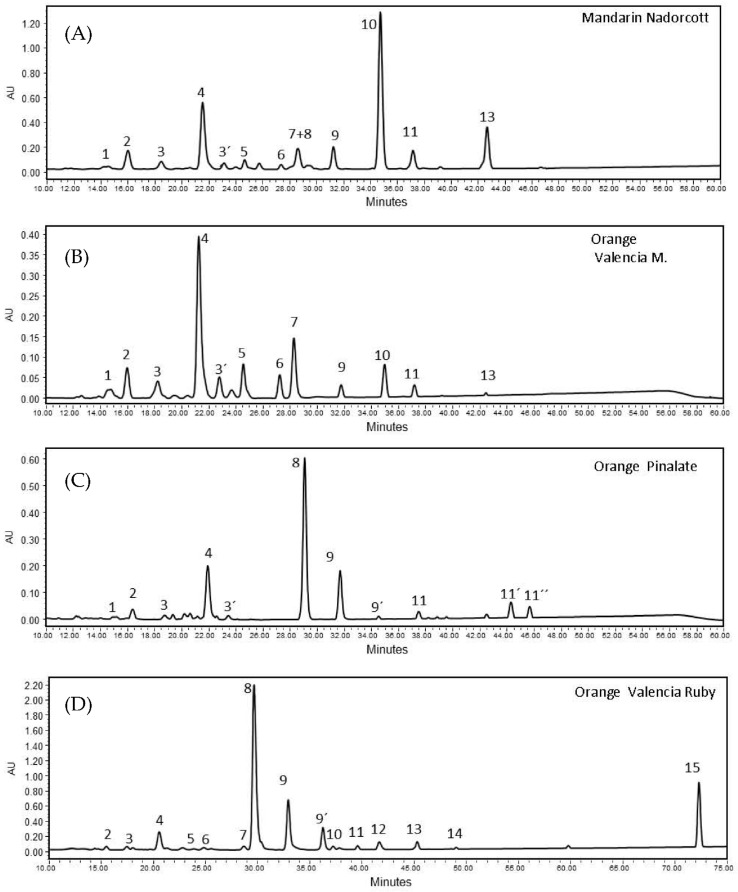
HPLC chromatograms of carotenoid extracts from pulps of (**A**) ‘Nadorcott’ mandarin (peak n^o^10 corresponds β-cryptoxanthin); (**B**) ‘Valencia late’ orange (peak n^o^4 corresponds to 9-Z-Violaxanthin); (**C**) ‘Pinalate’ orange (peak n^o^8 and 9, 9′ correspond to phytoene and two isomers of phytofluene, respectively); and (**D**) ‘Ruby’ Valencia orange (peak n^o^15 corresponds to lycopene). Identification of other carotenoid peaks are indicated in Supplementary Materials S2 Table S2. All profiles are MaxPlot chromatograms (each carotenoid shown at its individual λ maxima).

The carotenoid composition in pulp extracts of the ‘Nadorcott’ mandarin and ‘Valencia late’, ‘Ruby’ Valencia and ‘Pinalate’ sweet oranges showed a distinctive profile for each variety. Overall, 15 carotenoids were identified in the extracts (Supplementary Materials Table S2). Among the varieties selected in this study, Valencia late extracts contained the lowest level of total carotenoids (5.63 ± 0.06 µg/g FW), followed by the ‘Pinalate’ orange (24.51 ± 2.26 µg/g FW), the ‘Nadorcott’ mandarin (31.21 ± 1.07 µg/g FW) and the ‘Ruby’ Valencia orange with the highest total carotenoids (111.69 ± 9.41 µg/g FW) (Table 1). Then, the four selected varieties evidenced very distinguished carotenoid/xanthophyll compositions (Table 1). The colorless carotenes phytoene and phytofluene account for approximately 75% and 13%, respectively, of total carotenoids in the ‘Pinalate’ and ‘Ruby’ fruits. However, the pulp of the ‘Ruby’ fruits accumulated about five times more of these carotenes than that of ‘Pinalate’ (Table 1). Moreover, ‘Ruby’ oranges were the only variety containing the red carotene lycopene (6.55 ± 0.51 μg/g FW), accounting for 6% of total carotenoids (Table 1). On the other hand, Valencia oranges accumulated mainly xanthophylls (95.5% of total carotenoids) such as violaxanthin (34%), anteraxanthin (14%) and β-cryptoxanthin (11%). The fruits of the ‘Nadorcott’ mandarin presented the highest concentrations of β-cryptoxanthin (46.8% of total carotenoids) among the four citrus varieties studied, and minor amounts of other xanthophylls and linear carotenes (Table 1). Figure 2 presents examples of the chromatograms obtained with the citrus fruits studied here (specific retention times of major peaks, R_t_, were registered in the chromatograms). 

### 3.2. Preliminary Oxidation Assays

After the preparation of egg yolk phosphatidylcholine (PC) and AML liposomes, both with 1.5 mM total lipid concentration, we performed oxidation assays to compare these liposomal systems in terms of oxidation susceptibilities. Supplementary Materials S3 presents the preliminary auto-oxidation assays of PC and AML in the absence of Fenton reactants but monitored by the free radical-sensitive probe C_11_-BODIPY^581/591^. The auto-oxidation of liposomes is promoted by atmospheric air dissolved in an aqueous solution. The areas above curves (AAC, inset Supplementary Materials S3) estimate the extension of lipid oxidation; Supplementary Materials S3 shows that AML were approximately 40% more spontaneously oxidized by atmospheric O_2_ than PC liposomes (*p* < 0.05).

Regarding the lipid/membrane oxidation induced by HO^•^ radicals (Fenton reaction; Equation (3), different concentrations of H_2_O_2_ (from 25 mM to 300 mM) and Fe^2+^:EDTA (from 25 μM to 2.25 mM, always keeping the 1:4 ratio with EDTA) were tested to obtain the appropriate concentration of HO^•^ radicals for liposome oxidation. Among several other tests, Supplementary Materials S4 shows: (A) 1.5 mM PC liposome oxidation induced by 100, 200 or 300 mM H_2_O_2_ with fixed 5 mM:20 mM Fe^2+^:EDTA; (B) 1.5 mM PC liposome oxidation induced by 25 mM H_2_O_2_ in the presence of different concentrations of Fe^2+^:EDTA (from 25 μM to 2.25 mM, 1:4 ratio with EDTA); and (C) log scales of half-time oxidations (t_1/2_ for 50% of lipid oxidation) in 1.5 mM AML and 1.5 mM PC liposome triggered by 25 mM H_2_O_2_ and different Fe^2+^:EDTA concentrations (1:4 ratio). As shown in Supplementary Materials S4C, AML liposomes present lower oxidation capacity upon increasing concentrations of Fe^2+^:EDTA than PCL liposomes, based on the t_1/2_ values calculated from C_11_-BODIPY^581/591^ kinetics. Therefore, we selected 25 mM H_2_O_2_ and 1.5 mM:6 mM Fe^2+^:EDTA (again 1:4 ratio) as the appropriate proportion of Fenton reactants to initiate lipid oxidation in further AML liposome assays in the presence of citrus carotenoids and/or ASC.

### 3.3. Preliminary Assays with Ascorbic Acid

Although the scavenging properties of the water-soluble ASC are relatively known [40], we decided to verify its mode of action in our novice 1.5 mM AML liposomal system. Therefore, a preliminary oxidation assay of 1.5 mM AML liposomes was performed in the presence of different ASC concentrations in an aqueous solution (from 20 μM to 20 mM). The lipid oxidation progression was again monitored by C_11_-BODIPY^581/591^, and HO^•^ radicals were alternatively produced here by mixing 25 mM H_2_O_2_ with 0.625 mM Fe^2+^ and 2.5 mM EDTA (1:4 ratio). By corroborating the high solubility of ASC in water and its high scavenging activity in aqueous milieu, it is clear to observe that increasing concentrations of ASC caused significant delays in the initiation of lipid oxidation, mentioned here as the ‘delayed lag phase’ (in min; Supplementary Materials S5). The lag phases of lipid oxidation in AML systems containing 20 μM, 0.2 mM, 2 mM and 20 mM of ASC were, respectively, 0.25 min, 13.1 min, 98.7 min and 153 min. Interestingly, ASC 20 mM completely prevented AML from lipid peroxidation initiated by HO^•^ radicals during the total 150 min period of measurement. Based on these results and on the endogenous concentration variability of ASC in citrus fruits [4], we decided to use the concentration range from 10 to 30 μM ASC (with fixed 10 μM citrus carotenoids) in further AML liposome assays.

**Figure 3:**
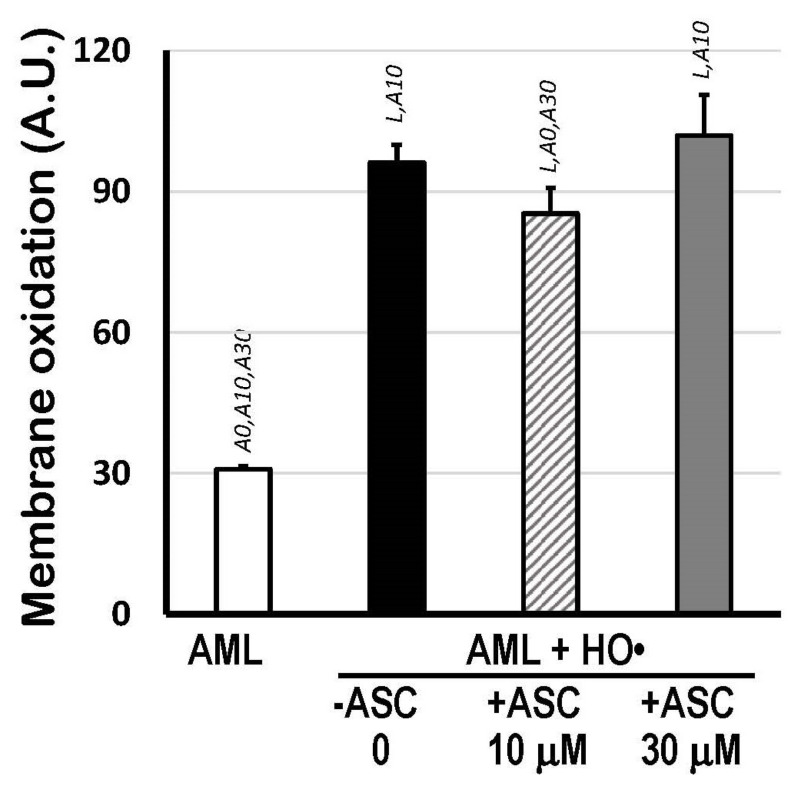
Membrane oxidation expressed as Areas Above Curves (AAC; A.U.) of C_11_-BODIPY^581/591^ decay kinetics indicating total lipid oxidation of 1.5 mM AML liposomes triggered by HO• radicals from Fenton reaction (25 mM H_2_O_2_ + 1.5 mM Fe^2+^/6 mM EDTA), in the presence or absence of 10 μM or 30 μM ASC, in PBS 50 mM, pH 7.5. Areas Above Curves (AAC) were calculated by integrating fluorescence intensity within the interval of 0 to 166 min. Statistical significance (*p* < 0.05) was indicated by symbols: L, compared to AML liposomes; A0, compared to (AML + HO•) system; A10, compared to (AML + HO• + ASC 10 μM) system; and A30, compared to (AML + HO• + ASC 30 μM) system.

The scavenging activity of 10 μM and 30 μM ASC (without carotenoids) against the HO^•^-mediated oxidation of 1.5 mM AML was also checked by calculating the areas above the curves (AAC) of C_11_-BODIPY^581/591^ kinetics, which represents the total extension of lipid oxidation from the moment Fe^2+^ ions (with EDTA, 1:4 ratio) were added. Figure 3 shows that only 10 μM ASC was able to protect AML liposomes (−15%; *p* < 0.05). No differences in AAC were observed between 30 μM ASC-treated AML and fully oxidized AML liposomes (without ASC).

At last, we performed a pilot assay focusing on the interaction of citrus fruits carotenoids (17 μM carotenoids from ‘Valencia’ sweet oranges) incorporated into 1.5 mM AML liposomes in the presence of a high concentration of ASC (2 mM) (Supplementary Materials S6). The lipid oxidation was initiated by the addition of 50 mM H_2_O_2_ and 2.5 mM Fe^2+^ with 10 mM EDTA (monitored by C_11_-BODIPY^581/591^). Interestingly, 17 μM Valencia orange carotenoids were only able to stop the progression of lipid oxidation approximately 30 min after the production of HO^•^ radicals in the aqueous solution, in the absence of ASC. On the other hand, 2 mM ASC, a 100-fold higher concentration compared to the carotenoids here, clearly prevented the oxidation of AML with an identical lag phase as reported before; approximately 100 min (Supplementary Materials S5). Unfortunately, we did not monitor fluorescence further (beyond 150 min) to verify if 17 μM carotenoids within AML liposomes could inhibit lipid oxidation that is already initiated (as shown in the absence of ASC; orange dots in Supplementary Materials S6).

### 3.4. Antioxidant Properties of Citrus Carotenoids with/without Ascorbic Acid

**Figure 4:**
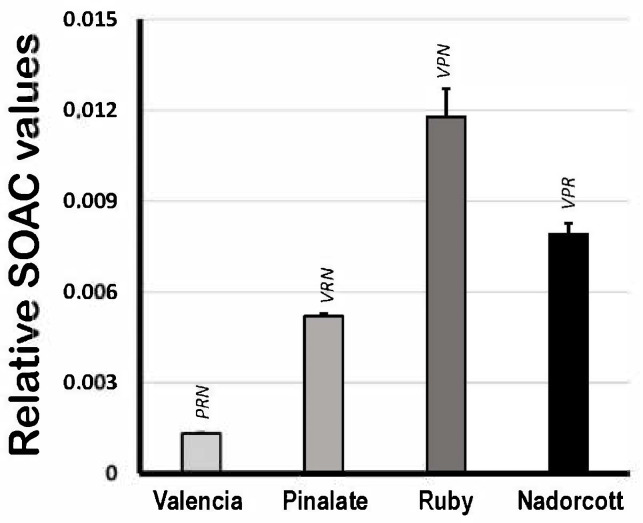
Singlet oxygen absorption capacity (expressed as relative SOAC values) of 10 μM carotenoids extracted from the pulp of the sweet oranges ‘Valencia late’, ‘Pinalate’ and ‘Ruby’ Valencia, and the mandarin ‘Nadorcott’ in ethanol/chloroform/water (49:50:1 *v*/*v*/*v*) solvent. Uppercase letters V, P, R and N indicate intergroup significant differences with, respectively, Valencia, ‘Pinalate’ and ‘Ruby’ oranges, and ‘Nadorcott’ mandarin (*p* < 0.05).

Figure 4 shows the quenching activity of citrus carotenoids in organic solvent (ECW solution) expressed as relative SOAC values, using α-tocopherol solutions as standards. Based on that, the lycopene-containing extracts from ‘Ruby’ oranges showed the highest SOAC values among samples (0.0118 ± 0.0009), followed by the ‘Nadorcott’ mandarin extracts with significant amounts of β-cryptoxanthin (0.0079 ± 0.0004). ‘The ‘Pinalate’ (0.0052 ± 0.0001) and Valencia pulp extracts (0.0013 ± 0.0001) showed proportionally lower quenching capacities against [O_2_(^1^Δ_g_)] than ‘Ruby’ and ‘Nadorcott’. The data presented here agree with previous results from our group published elsewhere [4]. It is worthy to note that these results reveal the inherent quenching capacities of citrus fruit carotenoid extracts against [O_2_(^1^Δ_g_)] generated in the organic phase, which is directly related to their conjugated polyene structure and other molecular-conformational aspects. These properties are expected to change in micellar systems, where water/lipid interfaces become a more relevant factor.

**Figure 5:**
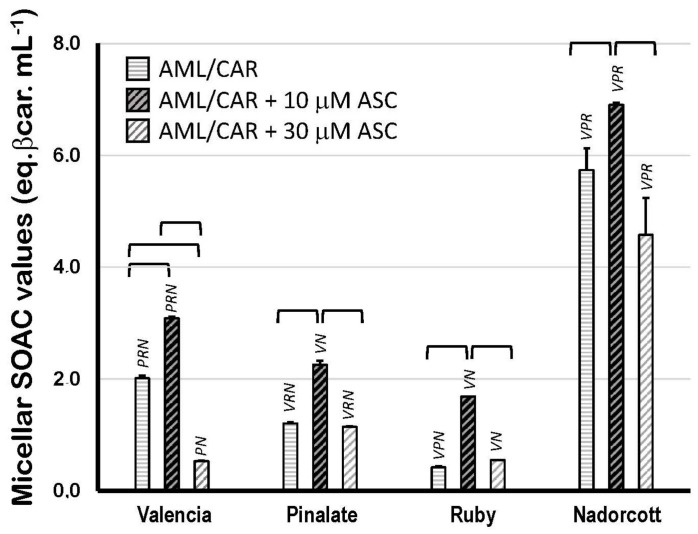
Singlet oxygen absorption capacity (expressed as equivalents of β-carotene/mL) of 10 μM carotenoids extracted from the pulp of the sweet oranges ‘Valencia late’, ‘Pinalate’ and ‘Ruby’ Valencia, and the mandarin ‘Nadorcott’ in 5% Triton X-100 micelles in PBS 50 mM, pH 7.5. Brackets indicate intragroup significant differences (*p* < 0.05); upper lock letters V, P, R and N indicate intergroup significant differences with, respectively, ‘Valencia’, ‘Pinalate’ and ‘Ruby’ oranges, and ‘Nadorcott’ mandarin (comparing the same treatment w/wo 10 μM or 30 μM ascorbate; *p* < 0.05).

Figure 5 displays the SOAC values of citrus fruit carotenoids in 5% Triton X-100 micelles (in 50 mM PBS, pH 7.5) in the presence of 10 or 30 μM ASC. The results show that carotenoids from ‘Nadorcott’ extracts have the highest quenching activity (5.74 ± 0.39 eq. βcarot/mL; Figure 5) against [O_2_(^1^Δ_g_)] when carotenoids are incorporated into micelles, where they are capable of interacting with the aqueous milieu at the water/lipid interface. In Triton X-100 micelles, carotenoids from ‘Valencia’ orange extracts showed a two-fold higher quenching activity than β-carotene itself, used as standard (2.01 ± 0.05 eq. βcarot/mL). Even though the ‘Pinalate’ and ‘Ruby’ extracts contained the highest levels of total carotenoids (mostly linear carotenes), both varieties showed the lowest quenching activities in 5% Triton X-100 micelles (1.20 ± 0.03 and 0.42 ± 0.02 eq. βcarot/mL, respectively). Interestingly, the addition of 10 μM ASC significantly increased the quenching activity of the carotenoid extracts from all citrus fruits, but predominantly in those with lower SOAC values in the absence of ASC: ‘Valencia’ orange (+53%), the ‘Pinalate’ variant (+87%), ‘Ruby’ (four-fold higher) and ‘Nadorcott’ mandarins (+20%). This effect was abolished or simply diminished when 30 μM ASC was added to the micellar systems. Unquestionably, this is an intriguing and worth investigating aspect of the molecular interactions between key water- and lipid-soluble antioxidants and their synergism/antagonism against lipid oxidation in micellar models of biological membranes.

**Figure 6:**
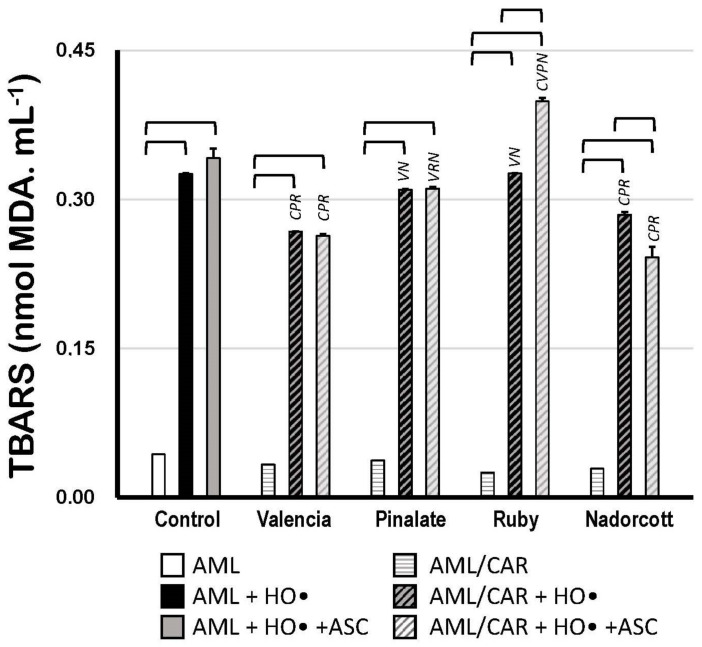
Lipid oxidation of 1.5 mM AML liposomes loaded or not with 10 μM carotenoids (AML and AML/CAR, respectively) extracted from the pulp of the sweet oranges ‘Valencia late’, ‘Pinalate’ and ‘Ruby’ Valencia, and the mandarin ‘Nadorcott’, triggered by HO• radicals from Fenton reaction (50 mM H_2_O_2_ + 3 mM Fe^2+^/12 mM EDTA), in the presence or absence of 10 μM ASC in PBS 50 mM, pH 7.5. Lipid peroxidation was expressed in nmol MDA/mL. Brackets indicate intragroup signif-icant differences (*p* < 0.05); upper lock letters C, V, P, R and N indicate intergroup significant differences with, respectively, control, ‘Valencia’, ‘Pinalate’ and ‘Ruby’ oranges, and ‘Nadorcott’ mandarin (comparing the same treatment, w/wo ASC; *p* < 0.05).

Figure 6 shows the TBARS concentrations after the oxidation of carotenoid-loaded AML liposomes compared to unloaded oxidized AML liposomes. In the absence of 10 μM ASC, only the ‘Valencia’ orange (−18%) and ‘Nadorcott’ mandarin extracts (−14%) offered significant protection to AML liposomes against HO^•^-promoted oxidation. Interestingly, in the presence of 10 μM ASC, only the ‘Nadorcott’ extracts conferred protection to AML liposomes against oxidation (−21%); a significant effect was not observed for the ‘Pinalate’ and ‘Valencia’ varieties (compared to the results without ASC). Finally, 10 μM ASC showed an unexpected prooxidant effect in ‘Ruby’ carotenoid-loaded AML liposomes, with +31% higher levels of TBARS than ‘Ruby’-carotenoid AML liposomes without ASC (Figure 6).

**Figure 7:**
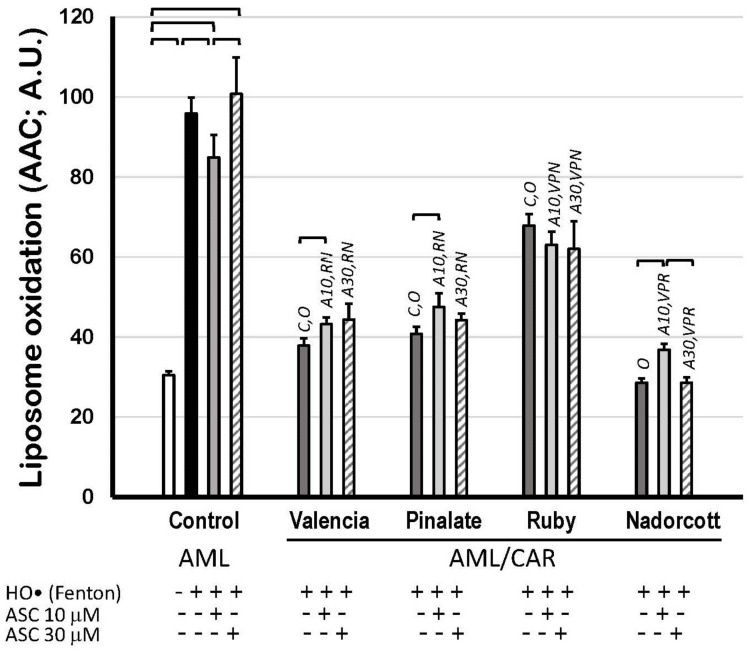
Lipid oxidation of 1.5 mM unilamellar liposomes with (AML/CAR) or without (AML) 10 μM carotenoids extracted from sweet ‘Valencia’, ‘Pinalate’ and ‘Ruby’ oranges, and ‘Nadorcott’ mandarin, triggered by HO• radicals (Fenton reaction: 25 mM H_2_O_2_ + 1.5 mM/6 mM Fe^2+^/EDTA), in the presence or absence of 10 μM or 30 μM ASC in PBS 50 mM, pH 7.5. Lipid peroxidation was expressed as Areas Above Curves (AAC) of C_11_-BODIPY^581/591^ kinetics (A.U.) during 180 min. Brackets indicate intragroup significant differences (*p* < 0.05); upper lock letters C, O, V, P, R and N indicate intergroup significant differences with, respectively, control, oxidized control, ‘Valencia’, ‘Pinalate’ and ‘Ruby’ oranges, and ‘Nadorcott’ mandarin (comparing the same treatment; *p* < 0.05). Upper lock codes A10 and A30 revealed significant differences compared to Control systems treated with ASC 10 μM and 30 μM, respectively (*p* < 0.05).

Finally, Figure 7 shows the scavenging properties of citrus fruit carotenoids in AML liposomes against precursor HO^•^ radicals but mostly against peroxyl (ROO^•^) and alkoxyl radicals (RO^•^), which were produced during the propagation steps of lipid oxidation. Supplementary Materials S6 illustrates that citrus carotenoids preferably retard the propagation of lipid oxidation (mediated by ROO^•^ and RO^•^) rather than preventing its initiation in an aqueous solution (by HO^•^). The extension of lipid peroxidation was expressed as AAC of C_11_-BODIPY^581/591^ kinetics, as described before (A.U.). These data show the robust protective effect of carotenoids within the lipid bilayer of AML liposomes, as most of citrus fruit extracts diminished lipid peroxidation by more than 30%. The lipid peroxidation inhibition by the ‘Valencia’, ‘Pinalate’ and ‘Ruby’ oranges and by the ‘Nadorcott’ mandarins, all in the absence of ASC, was: 60.5%, 57.5%, 29.2% and 70.2%, respectively. Neither 10 μM nor 30 μM ASC significantly affected the scavenging properties of citrus carotenoid extracts, except for a prooxidant effect (28.9%) of 10 μM ASC with ‘Nadorcott’ carotenoids (Figure 7).

## 4. Discussion

Citrus fruits have long been considered as one of the best sources of micronutrients for human nutrition, especially because of their contents of ascorbic acid (ASC), carotenoids and polyphenols/flavonoids [41]. ASC and carotenoids, apart from their function as enzymatic cofactors or regulators for immunological, sensorial and vascular functions (pro-vitamin A activities), exert key roles in redox metabolism as frontline dietary antioxidants to counteract molecular modifications promoted by ROS/RNS in cells [42,43]. However, although all these compounds present significant antioxidant properties per se, their effect in vitro and in vivo is often magnified when in combination, by mechanisms not yet fully understood. These synergistic or merely additive effects result in the optimized protection of cellular structures against oxidative insults that are the molecular basis of several human diseases [44]. Without discarding their role in protective redox-signaling cascades, e.g., the redox-sensitive cascades of Kelch-like ECH-associated protein 1 complex with nuclear factor erythroid 2-related factor 2 (Keap1-Nrf2) and nuclear factor kappa light chain enhancer of activated B cells (NF-κB) [45], at least part of the antioxidant effect of ASC and carotenoids is observed in the water/lipid interface of biological membranes, where exactly lipid-soluble antioxidants, such as carotenoids, tocopherols, and tocotrienols, interact with the water-soluble ones, like ASC, glutathione, and polyphenols/flavonoids [16,46,47].

Interestingly, in the lipid phase of biological membranes, mixed carotenes and xanthophylls (all comprising the whole family of carotenoids) apparently interact with each other to form a ‘tunneling system’ that efficiently transfers harmful free radicals—such as peroxyl (ROO^•^) and alkoxyl (RO^•^)—from the hydrophobic core of lipid bilayers to the water/lipid interface, where other water-soluble scavengers could transform them into non-radical products [47,48]. This mechanism was already described to explain the synergism between α-/γ-tocopherols and ASC, when inhibiting the oxidation of phospholipids in membranes [44]. However, the redox chemistry of both ASC and carotenoids has been long proven to be much more complicated, since these compounds also present pro-oxidant properties under some circumstances [43]. For example, carotenoids are prone to form very reactive peroxycarotenyl radicals (Car-OO^•^) under high oxygen tension, due to the additive reactions of molecular O_2_ with carotenyl radicals [48]. Furthermore, high concentrations of ASC enable the reduction in ferric ions (Fe^3+^) back to ferrous forms Fe^2+^, which are redox catalysts for the generation of harmful ROS/RNS [49,50]. 

### 4.1. Carotenoid Content in Citrus Fruits

The carotenoid content and composition found in the four varieties studied are similar to those described previously by other authors [4,9,21]. Although the extracts from citrus fruits are complex mixtures of different carotenes and xanthophylls, it is tempting to focus on the most prevalent carotenoids in each extract and attribute them to be at least part of the biochemical properties observed. Therefore, an interesting task was to compare the carotenoid content in the ‘Ruby’ and ‘Pinalate’ varieties, since both possess very high and similar relative contents of phytoene and phytofluene (respectively, 75% and 13%), but only ‘Ruby’ contains lycopene. The comparison between the ‘Valencia’ orange and ‘Nadorcott’ mandarin is relatively valid in the sense that both are mainly composed of xanthophylls, especially in terms of β-cryptoxanthin content (11% and 47%, respectively). It is also worth noting that the ‘Valencia’ orange also contains a substantial content of violaxanthin and antheraxanthin, while ‘Nadorcott’ presents higher levels of β-cryptoxanthin and violaxanthin in the extracts (Table 1). Overall, it would be reasonable to group these varieties as pairs regarding their carotenoid composition: ‘Pinalate’ and ‘Ruby’ oranges, with large contents of colorless carotenes; and the ‘Valencia’ orange and ‘Nadorcott’ mandarin, having higher proportions of xanthophylls. 

### 4.2. Oxidizable Properties of AML 

Although we used here a relatively novel biomimicking system for animal cell plasmatic membranes, this research was not focused on the characterization of the physicochemical properties of the AML liposomes but on understanding the antioxidant properties of citrus carotenoids and ASC in biological membranes (or their analogs). Nevertheless, a preliminary analysis of the oxidizable properties of the novel AML liposomal system was required. Therefore, we compared our AML system to PC liposomes and showed that AML is more autoxidizable than PC liposomes in the absence of Fe^2+^ ions (Supplementary Materials S3). PC liposomes showed higher rates of lipid oxidation with increasing Fe^2+^ concentrations compared to AML liposomes (Supplementary Materials S4C). The cholesterol content in AML, together with sphingolipids and ethanolamine-based phospholipids, possibly creates lipid micro-aggregates called ‘lipid rafts’, where fluidity, lipid compaction and even H^+^/OH^−^ permeation are altered [51]. Such structural peculiarities would obviously incur in distinguished oxidizable properties of AML systems compared to the more homogeneous PC liposomes. Moreover, lipid bilayers containing cholesterol at physiological levels (as the AML systems here) were shown to protect lipid-anchored fluorescent probes from oxidation by HO^•^ radicals (from Fenton’s reaction), probably due to the same structural lipid arrangements in ‘lipid rafts’, as mentioned before [35]. The oxidizable properties of AML were explored and preliminary assays established the fixed concentrations and proportions between Fenton’s reactants for efficient HO^•^ radical production here: 25 mM H_2_O_2_ and 1.5 mM Fe^2+^ with 6 mM EDTA, which is sufficient for substantial AML oxidation in experimental assays (Supplementary Materials S4B).

Different experimental conditions, but still physiologically relevant, were also tested to verify how ASC or carotenoids from citrus fruit extracts could act as antioxidants in our liposomal AML systems. As shown in Supplementary Materials S5 and S6, (i) the water-soluble antioxidant ASC retards the initiation of lipid oxidation in AML liposomes in a non-linear dose-dependent manner (Supplementary Materials S5); and (ii) carotenoids within the lipid bilayers of AML liposomes cannot block the initiation of lipid oxidation, but, rather significantly inhibit the progression of the chain reaction in AML systems (Supplementary Materials S6). These putative mechanisms were partially elucidated based on experiments performed with higher concentrations of reactants and antioxidants than those established by the standard assays here (Supplementary Materials S4B). Finally, under standardized oxidation conditions—25 mM H_2_O_2_ and 1.5 mM Fe^2+^ with 6 mM EDTA, as aforementioned—10 μM ASC indeed showed a slight (−15%) protective effect against HO^•^-mediated oxidation in AML liposomes, whereas 30 μM ASC was not able to protect the liposomes anymore (Figure 3). Again, microenvironmental conditions, as those established by hypothetically formed ‘lipid rafts’ in AML, could affect the molecular interactions between Fe^2+^ ions and phospholipids at the water/lipid (AML) interface and then induce the already known pro-oxidant activity of the ASC [51]. Most of the pro-oxidative activity of ASC is due to the regeneration of the redox-active ferrous ions (Fe^2+^) for Fenton reaction by the reduction in Fe^3+^ ions, with the concomitant formation of ascorbyl radicals (ASC^•−^).

### 4.3. Antioxidant Interactions between ASC and Citrus Carotenoids

In fact, the antioxidant activity of citrus carotenoids could be divided into their ‘scavenging’ activity—the capacity to intercept aggressive free radicals to produce more electronically stable (and less reactive) species—and their ‘quenching’ activity—the capacity to suppress excitatory energy from [O_2_(^1^Δ_g_)] [14]. Altogether, both the scavenging and quenching activities of carotenoids diminish the overall extension of lipid peroxidation in biological membranes induced by ROS/RNS. 

Regarding the quenching activity of citrus carotenoids, the SOAC values in Figure 4 show that the very high levels of the colorless carotenes, phytoene and phytofluene, and the 6% of lycopene in the ‘Ruby’ oranges (Table 1) dramatically increased the quenching activity of carotenoid extracts, as it has been suggested for red-fleshed oranges [52], compared to ‘Pinalate’ (−56%) extracts that also showed relevant concentrations of the colorless carotenes (Table 1). ‘The ‘Valencia’ oranges (~90% lower) and ‘Nadorcott’ mandarins (−43%) also showed lower SOAC scores than ‘Ruby’ extracts. Not surprisingly, lycopene was long considered as one of the most efficient [O_2_(^1^Δ_g_)] quencher carotenoids in biological systems [53]; more recently, relevant quenching properties were also associated with phytoene and phytofluene [54]. In citrus fruits, lycopene has also been proven to excel as an antioxidant by protecting citrus fruit peel from the oxidative damage induced during post-harvest cold stress [4,55].

Moreover, recent data have shown that violaxanthin, together with β-cryptoxanthin (the main carotenoids in ‘Valencia’ oranges and ‘Nadorcott’ mandarins), plays a major role in preventing [O_2_(^1^Δ_g_)]-mediated chilling injury in post-harvested fruits, with marginal participation of ASC [23]. All these conclusions were taken from the inherent capacity of the carotenoid extracts in organic solvent (ECW), which reflects a strict relation with the chemical structures of these carotenoids and their molecular interactions in organic solvents. When approaching real or natural conditions, we perceived that such inherent quenching behavior probably prevails within the hydrophobic core of natural membranes against [O_2_(^1^Δ_g_)] produced or present at that specific site. However, the water/lipid interface and vicinities are pivotal loci for the initiation of oxidative mechanisms and [O_2_(^1^Δ_g_)] formation, e.g., by the classic reaction between H_2_O_2_ and hypochloride (OCl^−^) in activated neutrophils and macrophages [56]. Exactly there, the water-soluble ASC action and the spatial disposition of carotenoids in membranes become relevant factors to explain the total potential of carotenoids as antioxidants [13,57].

Therefore, the SOAC assay was repeated in 5% Triton X-100 micelles (50 mM PBS, pH 7.5) in conditions that, at least in part, mimic the spatial distribution of carotenoids in a lipid phase. Moreover, micellar systems allow the observation of possible interactions between the water-soluble antioxidant ASC and carotenoids against [O_2_(^1^Δ_g_)]. In agreement with the preliminary oxidative assays in AML liposomes (Figure 3), 30 μM ASC diminished SOAC scores in micelles, independently of the composition of citrus carotenoids present (Figure 5). In this case, the ASC effect was not related to the reduction in ferric ions back to the active ferrous form (Fe^3+^→Fe^2+^), but possibly to molecular interactions with polar groups of citrus carotenoids and subsequent effects on fluidity and permeability of the lipid phase. Indeed, recent findings have shown that ASC (although necessary in the protonated form) has a significant permeability into lipid bilayers inflicting in the variant susceptibility of component lipids to oxidative injury [58]. Nevertheless, despite all the cautions taken avoiding metal contamination, we cannot exclude the possibility of the minimal presence of iron ions in the micellar system, which could trigger the prooxidant activity of ASC. On the other hand, 10 μM ASC was protective in all circumstances tested here, based on the micellar SOAC values (Figure 5). Interestingly, when working in micellar systems—which include the water/lipid interface and the spatial distribution of carotenoids within the lipid monolayer—the relative SOAC value of the β-cryptoxanthin-rich extract from the ‘Nadorcott’ mandarin was three-fold higher than that from the ‘Valencia’ oranges.

The previously observed patterns of SOAC values in organic solvents (inherent structural properties of carotenoids in extracts) were not reproduced under micellar conditions (Figure 7). The β-cryptoxanthin composition in mandarins reach almost 50% of the total carotenoid content in extracts (Table 1), which inputs its massive contribution to the quenching effect observed in micellar systems. β-Cryptoxanthin, also known as 3-hydroxy-β-carotene or β,β-caroten-3-ol, belongs to the class of oxygenated carotenoids known as xanthophylls. However, different from most of the xanthophylls, β-cryptoxanthin contains only one polar group (hydroxyl group) in one of the two edging β-ionone rings, which means that that HO-group probably interacts with polar groups of the water/lipid interface [59]. The rest of the molecule should be associated within the hydrophobic core through van der Waals interactions with the long carbonic non-polar chains of the surfactant. In general, carotenoids increase the hydrophobicity of the membrane interior, but these effects were stronger for dipolar xanthophylls, such as lutein, with moderate effects for ‘monopolar’ xanthophylls, such as β-cryptoxanthin, and negligible for non-polar carotenoids, like β-carotene [60]. Classic studies have already shown that β-cryptoxanthin was more effective than β-carotene (a highly hydrophobic carotenoid) against oxidation initiated both in the aqueous and lipid phases [61]. 

Regarding the scavenging activity of citrus carotenoids, both assays of HO^•^-triggered lipid peroxidation in AML liposomes (assayed as TBARS or C_11_-BODIPY^581/591^; Figure 6 and Figure 7) revealed approximately the same protective pattern in the absence of ASC concentrations: ‘Nadorcott’ mandarins > ‘Valencia’ oranges ~ ‘Pinalate’ oranges > ‘Ruby’ oranges. The extension of lipid peroxidation protection differs upon the method applied, keeping in mind the difference between the sensitivities of the TBARS assay and the C_11_-BODIPY^581/591^ method [62,63]. Moreover, despite the higher sensitivity of the fluorescent method, the incorporation of C_11_-BODIPY^581/591^ in liposomes was also associated with changes in some physicochemical properties of biological or synthetic membranes (in addition to the carotenoid effect), exposing these structures to exacerbated oxidative conditions [64]. In general, minor additional effects were observed in the presence of ASC at any tested concentration (Figure 6 and Figure 7). The unresponsiveness of ASC might reflect the distinct mode of action of the water-soluble ASC and lipid-soluble carotenoids in lipid bilayers, as demonstrated in preliminary assays (Supplementary Materials S6). These data showed that in our AML system, the interaction between ASC and carotenoids was not evident as we expected, since ASC was probably retarding the initiation of lipid oxidation in AML by blocking HO^•^ radicals in aqueous milieu (or at the water/lipid interface), whereas carotenoids in liposomes were alternatively blocking the progression of lipid oxidation after its initiation (Supplementary Materials S6).

Therefore, we were not able to observe any ‘synergism’ between ASC and citrus carotenoids against lipoperoxidation (by scavenging activity) in modeled AML liposomes. Major effects of ASC were observed in citrus carotenoids quenching effects against [O_2_(^1^Δ_g_)]. Nevertheless, it is tempting to compare all the antioxidant scores in order to establish a hypothetical pecking order for the ‘functional value’ of the citrus fruit analyzed here based on their antioxidant performances. Table 2 quantifies the observed antioxidant effects of citrus carotenoids extracts in all tests applied here (including the observed ASC-carotenoid interactions in micellar SOAC assays).

## 5. Conclusions

In the current study, we have evaluated the quenching and scavenging properties of citrus fruit carotenoid extracts in the presence or absence of ASC in organic solvents, in aqueous solutions, in micelles (models of lipid monolayer) and liposomes (lipid bilayers) that mimic animal cell membranes. Altogether, these results show that most citrus carotenoid extracts displayed significant and balanced antioxidant properties, alternating between their scavenging and quenching properties. However, extracts of the ‘Nadorcott’ mandarin presented an overall remarkable performance compared to that of other citrus varieties; at least part of these effects could be associated with the higher concentrations of β-cryptoxanthin present in the pulp of mandarins. This study reinforces the contribution of citrus fruits in promoting health to humans, mainly by offering a substantial composition of interactive antioxidants—carotenoids and ascorbic acid here—that could protect the cellular membrane against oxidative injury. 

## Figures and Tables

**Table 1:** Carotenoid composition (µg/g FW) and percentage (%) of individual carotenoids over the total content in the pulp extracts of ‘Nadorcott’ mandarin and Valencia late, ‘Ruby’ Valencia and ‘Pinalate’ sweet oranges. The amount of phytoene, phytofluene, luteoxanthin and z-carotene represents the sum of the different isomers identified. Traces indicates amounts lower than 0.05 µg/g FW.

Carotenoids	‘Nadorcott’ Mandarin		‘Valencia’ Orange		‘Pinalate’ Orange		‘Ruby’ Orange	
	**µg/g FW**	**%**	**µg/g FW**	**%**	**µg/g FW**	**%**	**µg/g FW**	**%**
Phytoene	3.28 ± 0.05	10.5	ND	-	18.25 ± 1.13	74.5	83.94 ± 6.54	75.2
Phytofluene	2.42 ± 0.12	7.9	0.20 ± 0.02	3.5	2.99 ± 0.77	12.2	14.58 ± 2.66	13.1
ζ-Carotene	1.46 ± 0.06	4.7	0.17 ± 0.01	3.0	1.13 ± 0.18	4.6	0.43 ± 0.02	0.4
Neurospor.	ND	-	ND	-	ND	-	1.40 ± 0.39	1.2
Lycopene	ND	-	ND	-	ND	-	6.55 ± 0.51	5.9
δ-Carotene	ND	-	ND	-	ND	-	0.35 ± 0.05	0.3
Lutein	0.51 ± 0.05	1.6	0.41 ± 0.01	7.2	0.11 ± 0.01	-	0.29 ± 0.01	0.3
β-Carotene	2.42 ± 0.39	7.8	traces	-	ND	0.4	0.71 ± 0.01	0.6
β-Crypx.	14.60 ± 0.60	46.8	0.64 ± 0.02	11.4	ND	-	0.50 ± 0.03	0.4
Zeaxanthin	0.70 ± 0.09	2.2	0.40 ± 0.01	7.2	0.08 ± 0.01	0.3	0.26 ± 0.01	0.2
Anterax.	0.56 ± 0.21	1.8	0.79 ± 0.02	13.9	ND	-	0.36 ± 0.06	0.3
*All-E*-Viol.	1.07 ± 0.01	3.4	0.35 ± 0.01	6.3	0.15 ± 0.01	0.6	0.24 ± 0.04	0.2
*9-Z*-Viol.	3.52 ± 0.15	11.3	1.94 ± 0.06	34.4	1.39 ± 0.12	5.7	1.69 ± 0.13	1.5
Luteox.	0.49 ± 0.01	1.6	0.53 ± 0.01	9.4	0.21 ± 0.03	0.8	0.38 ± 0.11	0.3
Neochrome	0.13 ± 0.01	0.4	0.19 ± 0.01	3.4	0.20 ± 0.02	0.8	ND	-
**TC**	31.21 ± 1.07		5.63 ± 0.06		24.51 ± 2.26		111.69 ± 9.41	

ND, not detected; TC, total carotenoids (µg/g FW).

**Table 2:** Relative quantification of the antioxidant effects of citrus fruit carotenoids extracts from ‘Nadorcott’ mandarins, and ‘Valencia’, ‘Pinalate’ and ‘Ruby’ sweet oranges based on their scavenging and quenching performances in vitro.

Citrus Fruit	BODIPY	TBARS	SOAC	SOAC_mic_	(+ASC) *	TOTAL
‘Valencia’	++	+	+	++	+	(7+)
‘Pinalate’	++	-	++	+	+	(6+)
‘Ruby’	+	-	+++	+	+	(6+)
‘Nadorcott’	+++	+	++	+++	+	(10+)

* Positive interactions with ASC in micellar SOAC assays. Signs represent: (-) minimal or no effect; (+) minor effect; (++) significant effect; and (+++) major effect.

## Data Availability

All the data are contained in the article and Supplementary Materials.

## Supplementary Materials

The following supporting information can be downloaded at: https://www.mdpi.com/article/10.3390/antiox12091733/s1, Supplementary Material S1 Figure S1: Logarithm fitting curves of DPBF absorbance decay (λ_max_ = 413 nm) by [O_2_(^1^Δ_g_)] in: (A) organic solvent (SOAC assay; see Materials & Methods), and (B) 5% Triton X-100 micellar suspension in PBS 50 mM, pH 7.5.; Supplementary Material S2 Table S2: Spectroscopic characteristics of main carotenoids identified in the chromatograms of pulp extracts of the four Citrus varieties; Supplementary Material S3 Figure S3: Auto-oxidation of 1.5 mM animal cell membrane-like liposomes (AML) compared to 1.5 mM egg-yolk phosphatidylcholine liposomes (PCL) monitored by the fluorescence decay at 600 nm of the free radical-sensitive probe C_11_-BODIPY^581/591^. Inset: Integrated Areas Above Curves (AAC) in PCL and AML to express total lipid/membrane oxidation within 0 to 166 min interval (* *p* < 0.05); Supplementary Material S4 Figure S4: HO^•^-mediated oxidation of 1.5 mM egg-yolk phosphatidylcholine liposomes (PCL) and/or 1.5 mM AML liposomes, monitored by the fluorescence decay at 600 nm of the free radical-sensitive probe C_11_-BODIPY^581/591^, in PBS 50 mM, pH 7.5, triggered by: (A) 100, 200 or 300 mM H_2_O_2_ in the presence of 5 mM:20 mM Fe^2+^:EDTA (1:4 ratio); (B) 25 mM H_2_O_2_ in the presence of different concentrations of Fe^2+^:EDTA (1:4 ratio) from 25 μM to 2.25 mM; and (C) log scales for comparison of oxidation in both PC and AML liposomes versus 25 mM H_2_O_2_ in the presence of different concentrations of Fe^2+^:EDTA (1:4 ratio) from 25 μM to 2.25 mM; Supplementary Material S5 Figure S5: Lipid oxidation of 1.5 mM AML liposomes, monitored by C_11_-BODIPY^581/591^ kinetics, and triggered by 50 mM H_2_O_2_ and 0.625 mM Fe^2+^ and 2.5 mM EDTA, in the presence of 20 μM, 0.2 mM, 2 mM, and 20 mM ASC, in PBS 50 mM, pH 7.5; Supplementary Material S6 Figure S6: Lipid oxidation of 1.5 mM AML liposomes loaded with 17 μM carotenoids from Valencia sweet oranges, monitored by C_11_-BODIPY^581/591^ kinetics, and triggered by 50 mM H_2_O_2_ and 0.625 mM Fe^2+^ with 2.5 mM EDTA (1:4 ratio), in the presence of 20 μM, 0.2 mM, 2 mM, and 20 mM ASC, in PBS 50 mM, pH 7.5.

## Abbreviations

AAC: area above curve; AML, animal cell membrane liposome; ASC, ascorbic acid; BHT, butylated hydroxytoluene; C_11_-BODIPY^581/591^, 4,4-difluoro-4-bora-3α,4α-diaza-*s*-indacene undecanoic acid; CTAB, cetyltrimethylammonium bromide; DAD, diode-array detection; DCM, dichloromethane; DPBF, 2,5-diphenyl-3,4-benzofuran; ECW, ethanol/chloroform/water solution; EDTA, ethylenediaminetetraacetic acid; EP, 1,4-naphthalene endoperoxide; EtOH, ethanol; FW, fresh weight; HPLC, high performance liquid chromatography; Keap1-Nrf2; Kelch-like ECH-associated protein 1 complex with nuclear factor erythroid 2-related factor 2; MDA, malondialdehyde; MeOH, methanol; MTBE, methyl *tert*-butyl ether; NF-κB, nuclear factor kappa light chain enhancer of activated B cells; PBS, phosphate-buffered saline; PC, phosphatidylcholine; PE, phosphatidylethanolamine; ROS/RNS, reactive oxygen/nitrogen species; SDS, sodium dodecylsulphate; SOAC, singlet oxygen absorbent capacity; TBARS, thiobarbituric acid-reactive substances; TEP, 1,1′,2,2′-tetraepoxypropane.
