# Supplementary material for: Antioxidant Interactions between Citrus Fruit Carotenoids and Ascorbic Acid in New Models of Animal Cell Membranes
Source: Antioxidants (Basel). 2023 Sep 7;12(9):1733. doi: 10.3390/antiox12091733 (PMC10525925; doi:10.3390/antiox12091733)

Supplementary Material S1. Logarithm fitting curves of DPBF absorbance decay (λ_{max} = 413 nm) by [$O_2(^1\Delta_g)$] in: (A) organic solvent (SOAC assay; see Materials & Methods), and (B) 5% Triton X-100 micellar suspension in PBS 50 mM, pH 7.5.

(A)

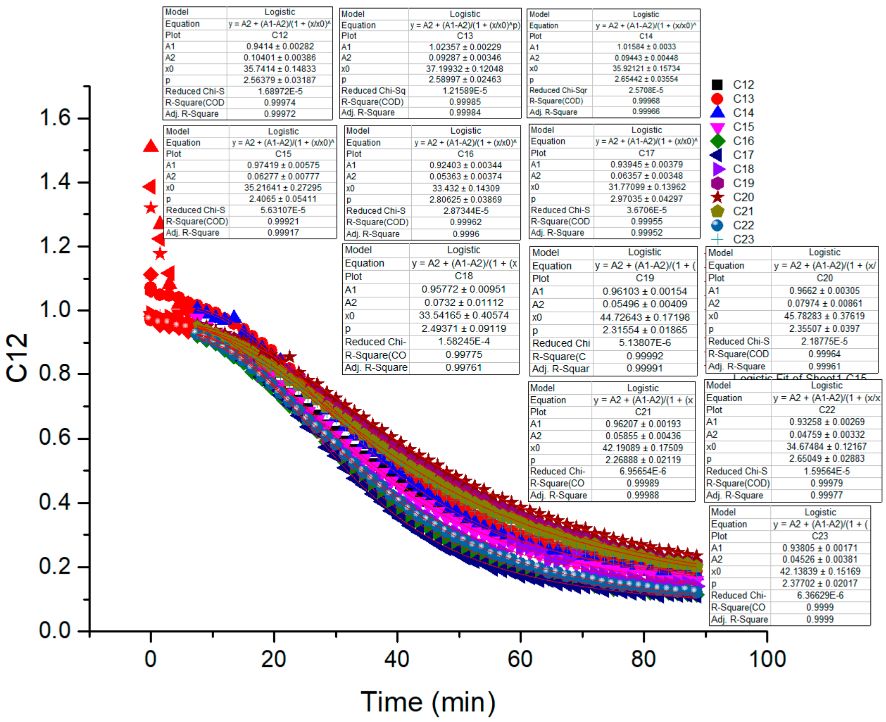

(B)

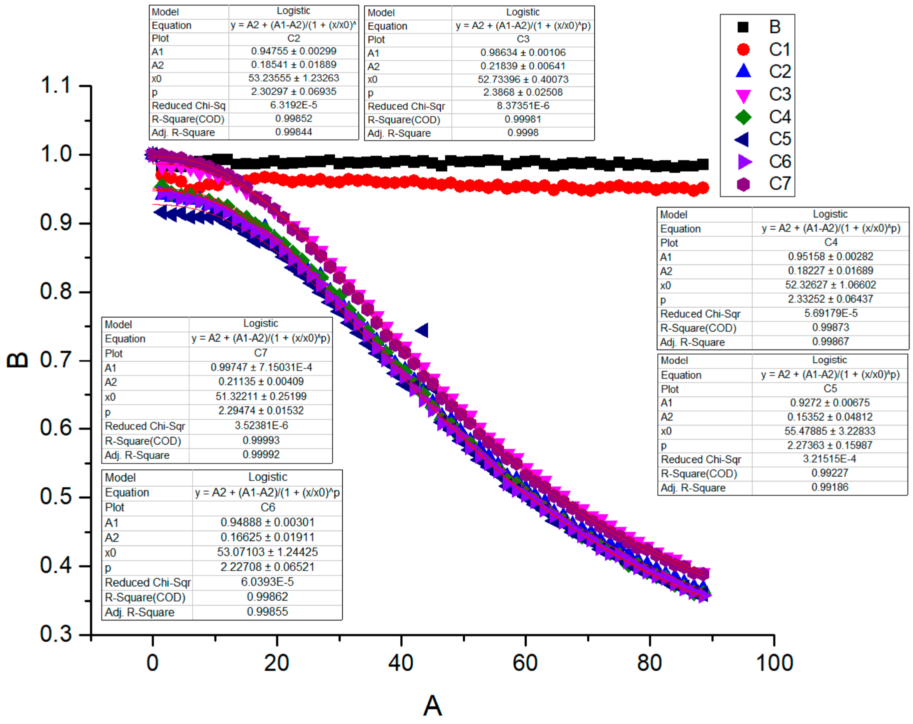

Supplementary Material 2. Table S2. Spectroscopic characteristics of main carotenoids identified in the chromatograms of pulp extracts of the four Citrus varieties.

Peak number	Carotenoid	UV-Vis absorption maxima (nm)
1	Neochrome	328(*Z*), 409, 431, 458
2	* all-*E*-Violaxanthin	415, 438, 468
3	Luteoxanthin isomer 1	396, 421, 448
3′	Luteoxanthin isomer 2	394, 417, 445
4	* 9-*Z*-Violaxanthin	328(*Z*), 412, 436, 464
5	* Lutein	s, 444, 472
6	* Zeaxanthin	s, 450, 475
7	* Antheraxanthin	s, 441, 469
8	* 15-*Z*-Phytoene	285
9	* Phytofluene isomer	331, 346, 364
9′	* Phytofluene isomer	s, 347, 363
10	* β-Cryptoxanthin	423, 450, 479
11	* tri-Z-ζ-Carotene	296(*Z*), 375, 399, 423
11′	* di-Z-ζ-Carotene	296(*Z*), 379, 400, 425
11′′	ζ-Carotene isomer	296(*Z*), 379, 400, 425
12	Neurosporene isomer	s, 433, 461
13	* β-Carotene	s, 452, 478
14	δ-Carotene isomer	431, 457, 487
15	* all-*E*-Lycopene	446, 472, 504

*Identified using authentic standards; s, shoulder.

Supplementary Material S3. Figure S3. Auto-oxidation of 1.5 mM animal cell membrane-like liposomes (AML) compared to 1.5 mM egg-yolk phosphatidylcholine liposomes (PCL) monitored by the fluorescence decay at 600 nm of the free radical-sensitive probe C_{11}-BODIPY$^{581/591}$. Inset: Integrated Areas Above Curves (AAC) in PCL and AML to express total lipid/membrane oxidation within 0 to 166 min interval (*p<0.05).

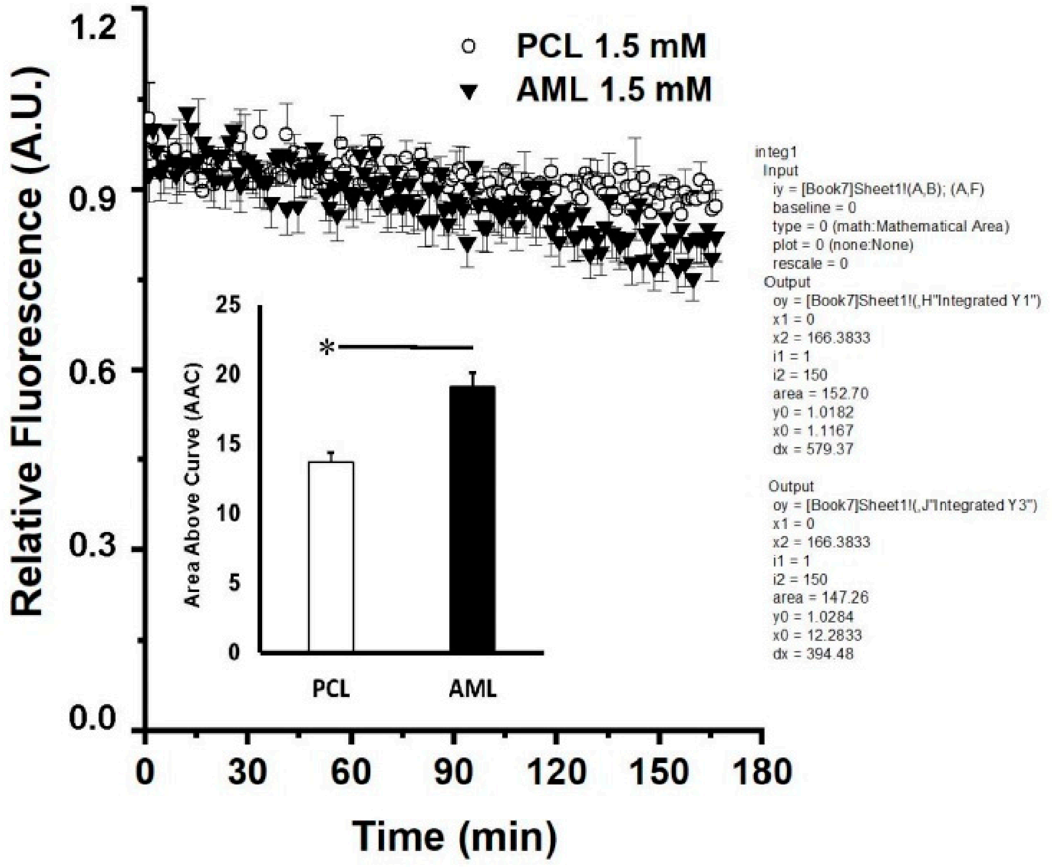

Supplementary Material S4. Figure S4. HO•-mediated oxidation of 1.5 mM egg-yolk phosphatidylcholine liposomes (PCL) and/or 1.5 mM AML liposomes, monitored by the fluorescence decay at 600 nm of the free radical-sensitive probe C_{11}-BODIPY$^{581/591}$, in PBS 50 mM, pH 7.5, triggered by: (A) 100, 200 or 300 mM H_2O_2 in the presence of 5 mM:20 mM Fe^{2+}:EDTA (1:4 ratio); (B) 25 mM H_2O_2 in the presence of different concentrations of Fe^{2+}:EDTA (1:4 ratio) from 25 µM to 2.25 mM; and (C) log scales for comparison of oxidation in both PC and AML liposomes versus 25 mM H_2O_2 in the presence of different concentrations of Fe^{2+}:EDTA (1:4 ratio) from 25 µM to 2.25 mM.

(A)

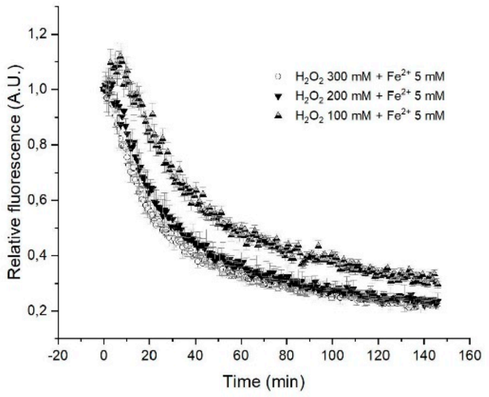

(B)

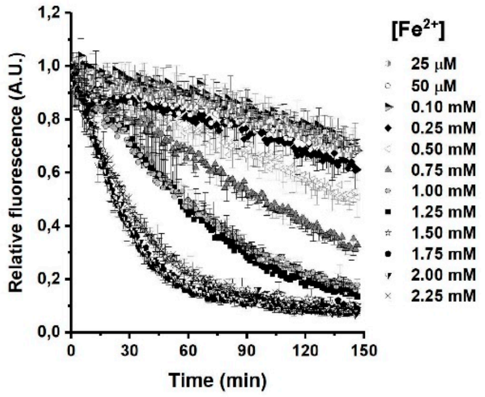

(C)

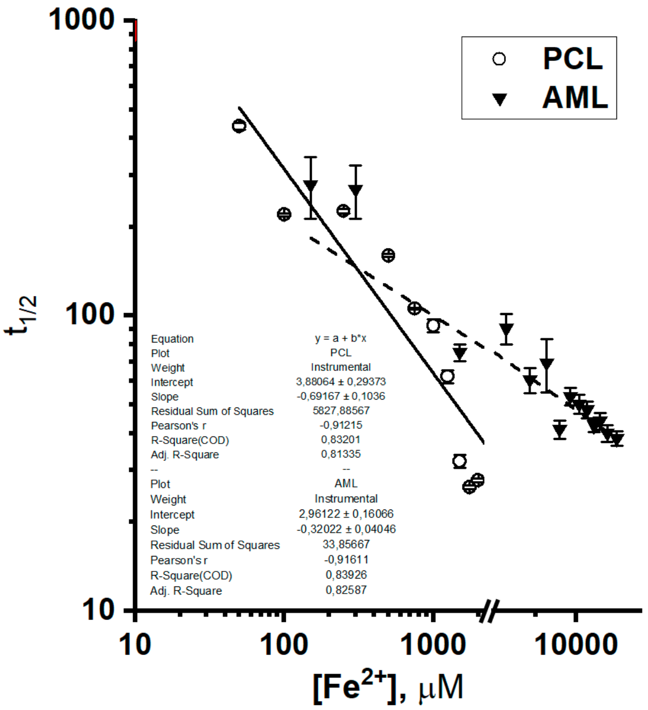

Supplementary Material 5. Figure S5. Lipid oxidation of 1.5 mM AML liposomes, monitored by C_{11}-BODIPY$^{581/591}$ kinetics, and triggered by 50 mM H_2O_2 and 0.625 mM Fe^{2+} and 2.5 mM EDTA, in the presence of 20 µM, 0.2 mM, 2 mM, and 20 mM ASC, in PBS 50 mM, pH 7.5.

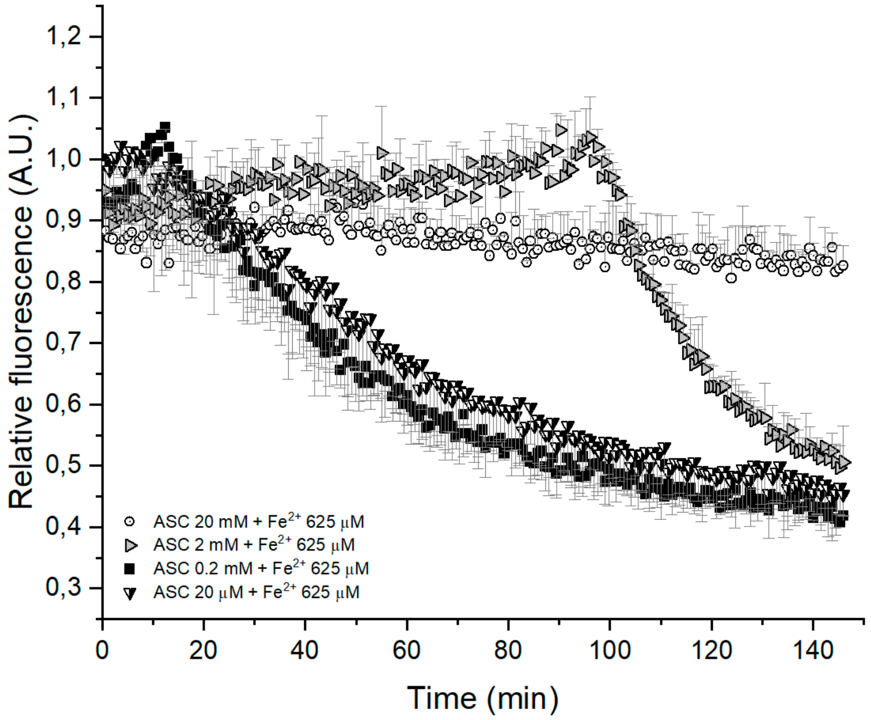

Supplementary Material 6. Figure S6. Lipid oxidation of 1.5 mM AML liposomes loaded with 17 µM carotenoids from Valencia sweet oranges, monitored by C_{11}-BODIPY$^{581/591}$ kinetics, and triggered by 50 mM H_2O_2 and 0.625 mM Fe^{2+} with 2.5 mM EDTA (1:4 ratio), in the presence of 20 µM, 0.2 mM, 2 mM, and 20 mM ASC, in PBS 50 mM, pH 7.5.

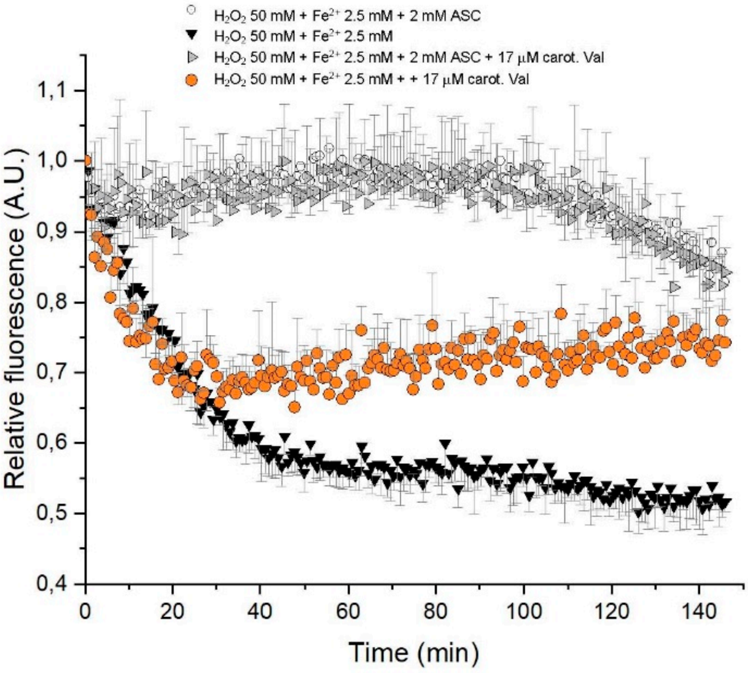

Supplement: Supplementary file 1 [file antioxidants-12-01733-s001.zip › antioxidants-2516159-SI.pdf]
